# Two opposite size effects of hardness at real nano-scale and their distinct origins

**DOI:** 10.1038/s41598-017-14734-w

**Published:** 2017-11-22

**Authors:** Rong Yang, Qun Zhang, Pan Xiao, Jun Wang, Yilong Bai

**Affiliations:** State Key Laboratory of Nonlinear Mechanics (LNM), Institute of Mechanics, Chinese Academy of Sciences, Beijing, 100190 People’s Republic of China

## Abstract

Although it has been well known that hardness of metals obtained with conical indenter remains a constant of about 3 times yield strength in conventional tests, and hardness will show a size effect of increasing hardness with decreasing indentation depth in micro-scale beyond 100 nm, the nano-indentation hardness experiments within 100 nm indentation depth usually show a large deviation and unclear trends. We report the cross-validated experimental and numerical results of two opposite depth-dependences of hardness at real nano-scale. That is to say, the indentation size effect (ISE) of hardness of single-crystal copper shows a rapid increase and then a slow decrease with increasing indentation depth within 100 nm depth. All of the results were coss-checked by means of both elaborated nano-indentation experiments with calibrated indenter tips and large scale molecular dynamics (MD) simulations. Further analysis of the MD results and experimental data reveal that the two opposite ISE of nano-hardness should be attributed to the finite roundness of the indenter tip and the intrinsic transition governing property of the material.

## Introduction

**Figure 1:**
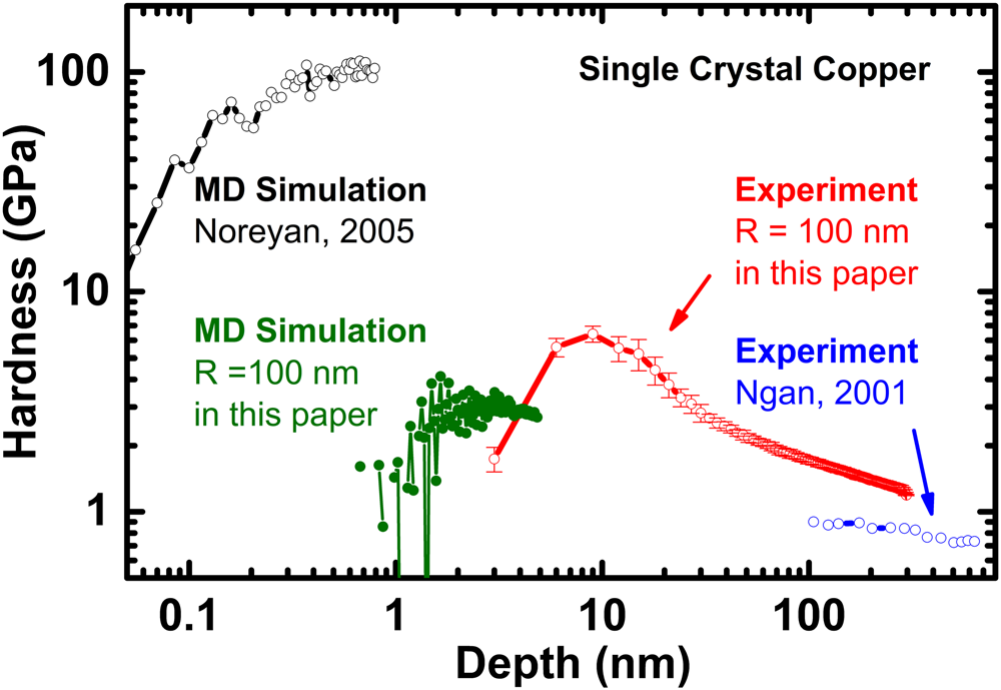
Comparison of numerical and experimental results of hardness in single crystal Cu, based on available literature and the results with tip radius of 100 nm obtained in this paper. The hardness trends are opposite in nano-scale (black) and micro-scale (blue). The simulation result (black) is two orders higher than the experiment one (blue). There is a gap in simulation and experiment between 1 nm to 100 nm from literature. The simulation (green) and experiment (red) results in this paper are in the same order and the trends are similar to each other, the hardness curves all go up then go down as the indentation depth deceases.

When refer to indentation size effect (ISE), the story would be the hardness increases as the indentation depth decreases. It is usually been taken as an example of “small is stronger”, and it has first been attributed to the accumulation of geometrically necessary dislocation (GND) and associated plastic gradients^1,2^. However, it is well known that the Nix-Gao^1^ model works fine for ISE at the submicron scale but needs some modification^3–5^ if the same model is used to interpret the data below 100 nm. Unfortunately, the experiment results are unreliable at this scale, and simulation results diverge from them too. From experiments, there are few experimental results to support whether ISE of hardness works at this real nano-scale (0.1 nm to 100 nm). The devil behind this is that in this depth range, hardness measurement take influences from the capacity of the instruments, noise from the environment, shape of the tip of indenter, besides the properties of the sample. Studies had shown work-hardening^6,7^, grain size and grain boundaries^8^, surface roughness^9,10^, and phase transformation^11,12^ will affect the harness measurement. As a consequence, the hardness-depth curves from indentation experiments at nano-scale show large deviation and the ISE is not clear in this range. The simulation^13^ result shown opposite ISE and the hardness value is two orders higher than that from experiments^14^, as shown in Fig. 1. There are accumulating evidences indicate that the indentation size effect (ISE) presents different tends compared to the conventional one in different types of materials, as in metallic glasses^15^ and fcc metals^16,17^. Thus, it is essential to carefully examine the ISE different from the conventional one at real nano-scale.

Here we provide results at real nano-scale, from experiments and simulations. Two opposite size effect of nano-hardness are observed, and the transition occours at indentation depth around 15 nm, both from experimental and numerical results, which show that the hardness goes up then goes down as the indentation depth decreases. The details of force-depth and hardness-depth curves from both approaches are compared. To match these two approaches, single crystal copper (Cu) with predetermined crystalline orientations was chosen to be investigated. To avoid above mentioned complications from indenter and sample, the shape of the indenter and the microstructure and surface roughness are carefully examined. Then MD simulations with up to 0.24 billion atoms are carried out, which took account of tip radius of the indenter, crystalline orientation and temperature. The hardness results from experiment and simulation are cross examined, and details of force-depth and hardness-depth curves are compared to reveal the physics behind ISE.

## Results

### Experimental results of single crystal copper indentation

**Figure 2:**
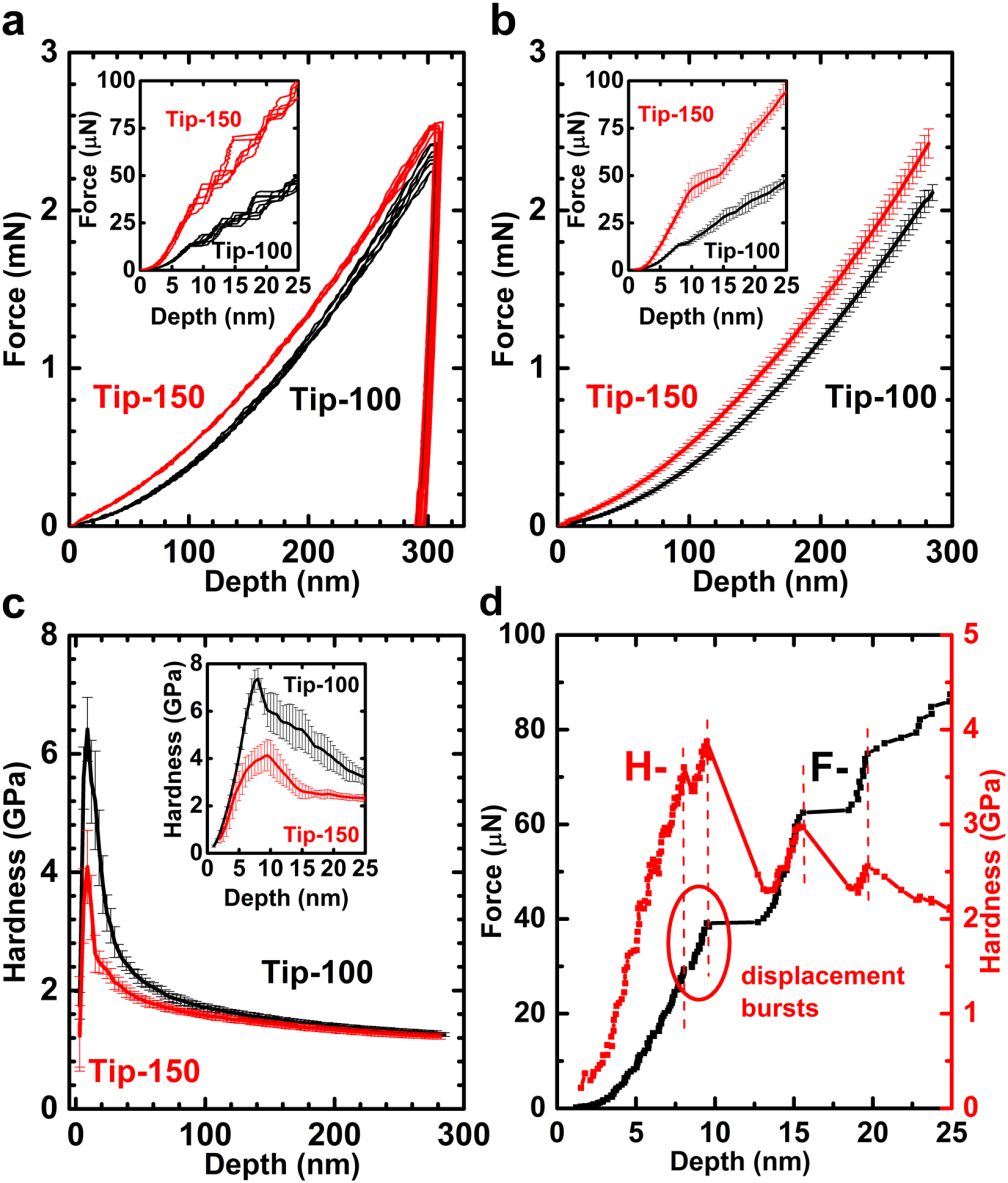
Experiment results of indentation on Cu. (**a**) Force-depth curves obtained using two indenters with 100 nm and 150 nm tip radius, respectively. The repeatability of the experiments is good when indentation depth is within 10 nm and over 25 nm. In the range of 8–25 nm, the curves separate due to displacement “bursts” patterns in the curves. The 150 nm tip loading curves are higher compare to these of 100 nm tip. (**b**) Average and error bar of force-depth curves from Tip-100 and Tip-150 (n = 18 tests) result in the same conclusion for the repeatability of the experiments (error bar: s.d.) and the influence of the tip radius. (**c**) Hardness curves of the Cu samples. The hardness goes up then goes down as the indentation depth deceases, the transition occurs in the range of 8–10 nm, and peak value can be observed in this range too. Hardness value is higher for Tip-100, the corresponding depth to the hardness peak is lower than that of Tip-150. The effect of the tip radius abates when the indentation depth is over 100 nm. (**d**) Displacement “bursts” in the loading curves. They correlate to each drop in hardness curve.

The single crystal copper (Cu) is used in our study, here the results of (100) Cu samples are presented, the results of other two orientations can be found in Supplementary document. The detailed processing in determining the surface roughness and orientations of the Cu samples and the shape of the indenter tips, as well as the indentation experiments are given in the Methods section. A status of low in both surface roughness (average roughness *Ra* in the range of 0.170~0.261 nm) and misorientation to the presumed crystalline direction (in the range of 0.5~1.5 degrees) are realized, which results the experimental curves to be with a small deviation. Two indenters are used in the tests and the radiuses are found to be approximate 100 nm (for Tip-100) and 150 nm (for Tip-150). The force-depth curves in Fig. 2a shows not only the repeatability of the experiments but also the influences of the tip radius. The influence of the tip radius to the loading curves can be found in Fig. 2a and b, which results in a higher load for tip with larger radius. The trend of hardness over indentation depth can be readily observed in Fig. 2c, which indicates that there are two opposite ISEs exist at nano-scale, since instead of continuing to go up as the indentation depth decreases, the hardness value decreases when depth is less than 10–15 nm. The influence of the tip radius can be distinguished in Fig. 2c. Although the load is higher when using Tip-150 when reaching the same depth, the hardness value (and the peak value) is lower for Tip-150. The Tip-100 curve reaches the hardness peak at shallower indentation depth. When the indentation depth is over 100 nm, the effect of tip radius abates and the two hardness curves get closer to each other, since more material is in contact with the pyramid part of the indenter, and the hardness value approaches to a constant, same as that from conical/pyramid indenter. A close look at each force-depth curves reveals that displacement “bursts” patterns in the loading curves, and each plateau in the force-depth curves results in the hardness decreases, which can be readily observed in corresponding hardness-depth curves in Fig. 2d. The modulus of Cu does not exhibit apparent size effect, as seen in Supplementary Figs 4–7.

### Numerical results of single crystal copper indentation

**Figure 3:**
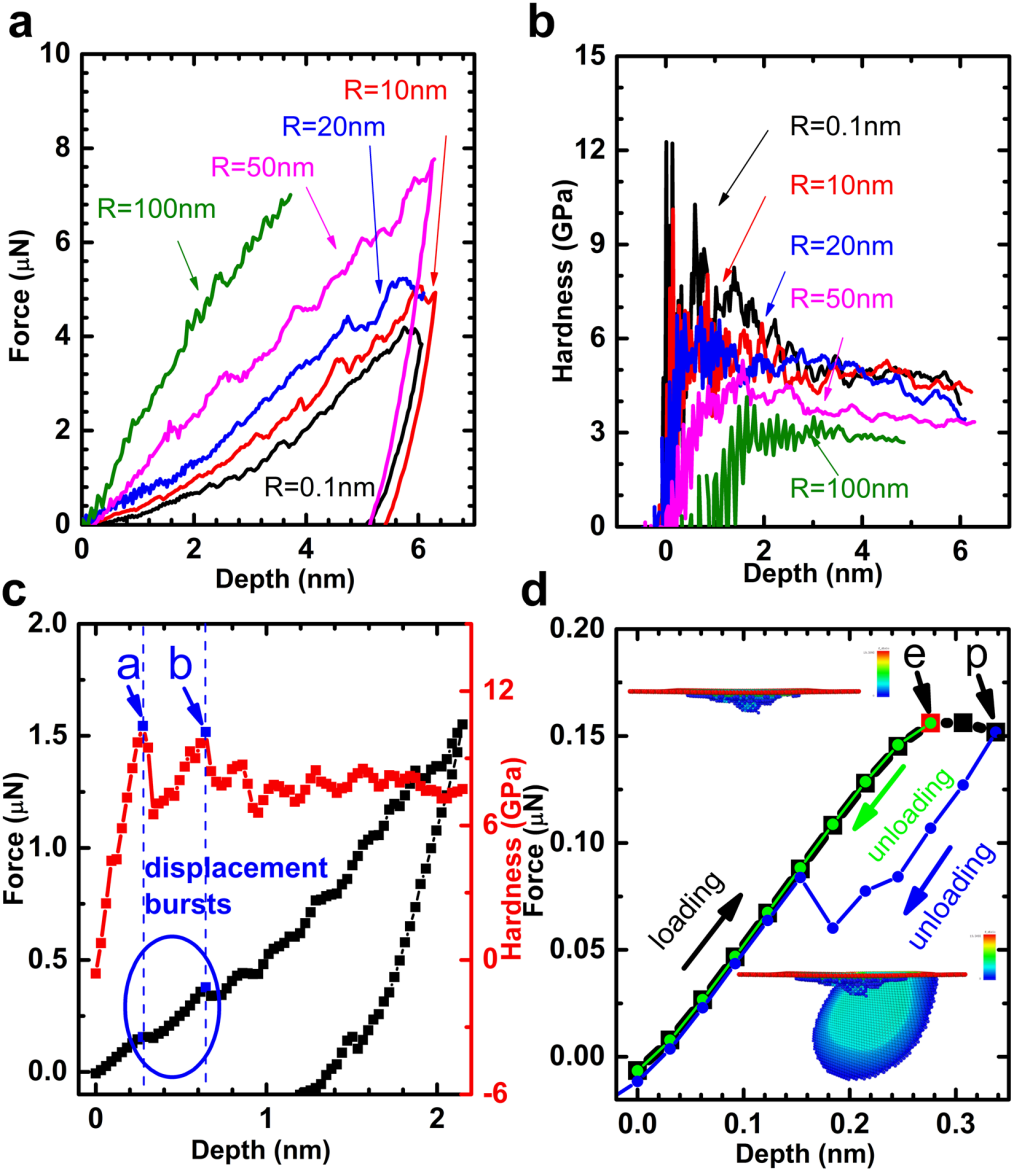
Simulation results of indentation on Cu. (**a**) Force-depth curves from MD simulations of 0.1, 10, 20, 50 and 100 nm radius tip. The load is higher with larger tip radius. (**b**) Hardness curves of the Cu. The hardness is lower with larger tip radius. (**c**) Displacement “bursts” patterns in the loading curves for the case of R = 10 nm at 0 K. This is similar to that from the experiments in Fig. 2d, and each drop of hardness value coincides with a burst in displacement curve. (**d**) A close look at the loading and unloading curves of Fig. 3c at shallow depth, and CSP distributions after the indenter is retrieved from the sample at step point e and p. The loading and unloading curves converge with each other before step point e, and CSP map shows the distortion of the lattice is not great and after the indenter is removed, the lattice will recover to origin state; and then a hysteresis loop emerges when loading beyond step point e, indicating the deformation is not reversible. CSP map shown stacking fault and dislocation initiate, and the lattice cannot be restored to its origin state after the indenter retrieves from the sample.

To understand the mechanism of the two opposite size effects, MD simulations are carried out to verify of the trend of nano-hardness behavior, as well as the effect of tip radius. The detailed in setting up the simulations are given in the Methods section. The cases with tip radius R = 0.1, 10, 20, 50 and 100 nm, Cu crystalline orientation of (111), (110) and (100), and temperature of 0 K and 300 K, are simulated, noting that R = 0.1 nm actually stands for the case of sharpest indenter, which is with one carbon atom (radius of 0.091 nm) at the tip of the indenter. The results of (100) orientation at 300 K are presented in Fig. 3a and b. The curves follow the same rules as the experiments, for larger indenter tip radius, the load is higher, but the hardness is lower. displacement “bursts” patterns in the loading curves can be observed in the loading curves (as shown in Fig. 3c for the case of R = 10 nm at 0 K), which is the same as that from experiments. Each step in the loading curve results in a decrease in the hardness curve, as illustrated in Fig. 3c of step points a and b. The first hardness drop occurs at about 0.3 nm, it corresponds to the emergence of plastic deformation in the material, as shown in Fig. 3d. Before reaching step point e, unloading curves coincides with the loading curves, and the central-symmetric parameters (CSP) shows that the distortion of Cu lattice is recovered after the indenter is retrieved from the surface, and the material exhibits “elastic” behavior. When loading beyond step point e to point p, the dislocations nucleate and propagate, the loading and unloading curves forms a loop which indicates “plastic” behavior, and the CSP map shows a dislocation surface grow and a dislocation loop is formed.

### Comparison of results from experiment and simulation

**Figure 4:**
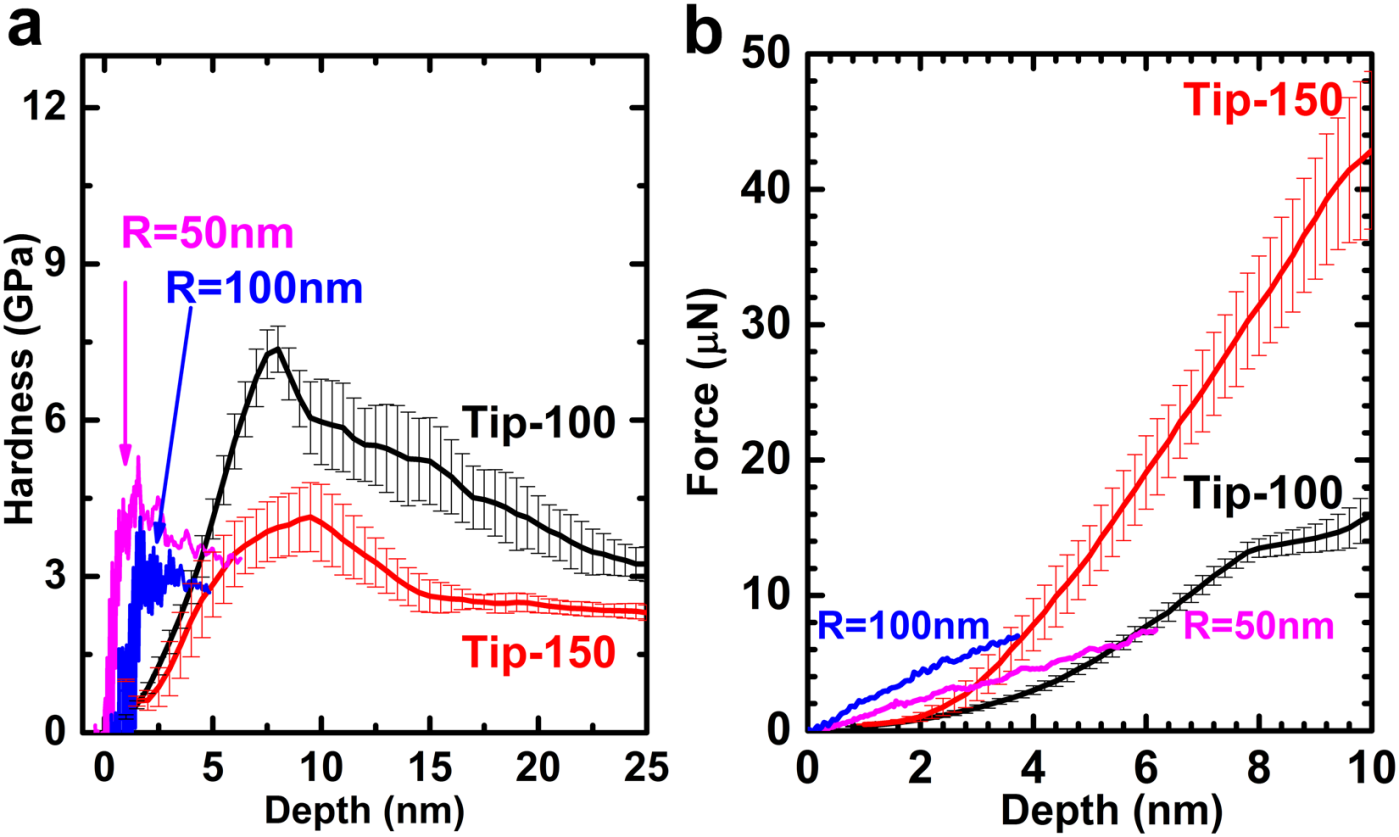
Comparison of the hardness and force results from experiment and simulation. (**a**) Hardness curves from Tip-100 and Tip-150 (experiment) is higher than those from R = 50 nm and 100 nm (simulation), and the depth to reach the hardness peak is larger as well. For the same tip radius (R = 100 nm), hardness from experiment is twice of that from the simulation. However, larger tip radius results in lower hardness and larger depth to reach the hardness peak is mutual in experiment and simulation. (**b**) Force-depth curves from experiment and simulation. Force curve from experiment is lower compares to that from the simulation. For the same tip radius (R = 100 nm), force from experiment is twice of that from the simulation. The influence of the tip radius is the same in both approaches.

Similarities can be observed between the results of both approaches, (a) The hardness curves increases then decreases when indentation depth decrease, the transition depth is within 25 nm, and the hardness peak value in the magnitude of 10 GPa; (b) Larger radius of the indenter tip results in higher load but lower hardness at the same depth, it also results in a lower hardness peak and a larger depth to reach the hardness peak; (c) Displacement “bursts” patterns can be observed in loading curves, and they correspond to the drops in the hardness curves. Discrepancies can be found in the actual hardness peak value and the depth to reach it, the hardness is higher in the results of experiments, although the load is higher in the simulations as shown in Fig. 4a and b. The explanation to this will be given in discussion.

## Discussion

**Figure 5:**
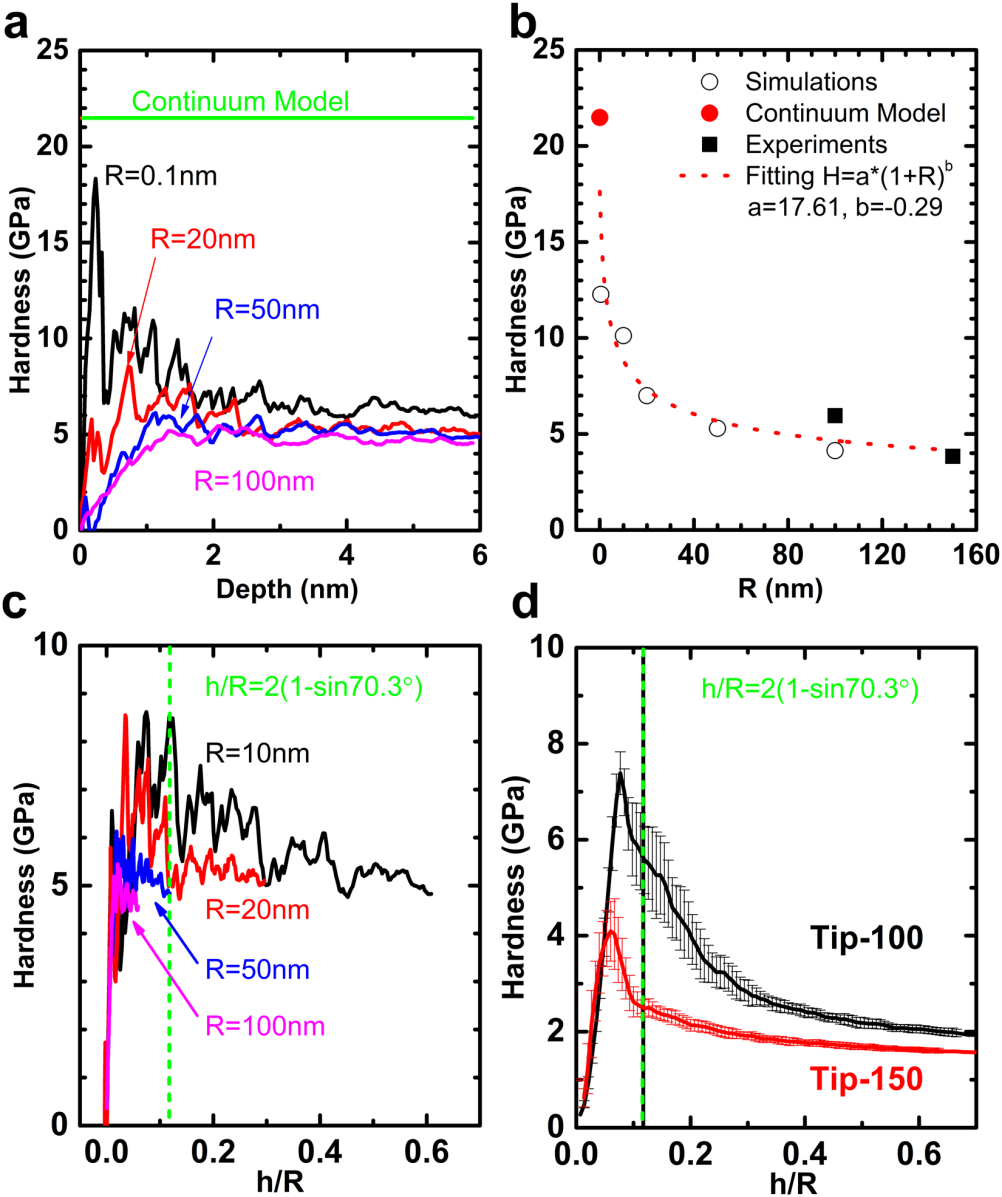
The influence of tip radius to the hardness. (**a**) Hardness curves from simulation and comparison with the theoretical hardness of conical indenter indents into elastic material. This value from continuum model stands for the upper limit of the hardness peak, equivalent to the result of R = 0 nm. (**b**) Hardness peak value over the tip radius. The results from experiment (black square dots), simulation (black round circles) and analytical (red round dot) seem follow the same trend, a power-law fitting curve (red dotted-line) to the data is plotted in the figure. The simulation and experiment results overlap at R = 100 nm. (**c**) The hardness curves from simulation (at 0 K) in terms of relatively depth (h/R). When h/R is small, the curves coincide with each other. The hardness transition occurs in spherical part of the indenter, as the hardness peaks are on the left side of the green line which indicates the boundary of spherical and conical part of the indenter. (**d**) The hardness curves from experiment in terms of relatively depth (h/R). Same observations can be found as these from the simulation, although the hardness and depth for the transition are higher.

The present study reveals that the conventional ISE in crystal Cu samples breaks down and two opposite ISE emerges at real nano regime. Specifically, the hardness will decrease when continuing to decrease the indentation depth after a certain depth range (2–25 nm), which is related to the radius of the indenter tip. The hardness peak value decreases as the tip radius increase. From conventional point of view, the shape of the indenter is ideally-perfect cone or pyramid, thus the hardness value will be a constant if the materials follow the classic continuum theories. For elastic case, according to the work of Sneddon^18^, the hardness will be

1 $$H=\frac{\cot \,\alpha }{2}{E}_{r}$$

where *α* is the half included-angle of the indenter which is 70.3°, and reduce modulus ${E}_{r}=E/(1-{\nu }^{2})$, for single crystal Cu, it will be about 120 GPa, as its elastic modulus around 110 GPa and Poisson’s ratio as 0.3. The hardness value will come to 21.48 GPa, as shown in Fig. 5a, it represents the upper limit of the hardness value of Cu. For plastic cases, our previous work^19,20^ showed, the hardness value will also be a constant, however, its value will be lower value than that from Eq. (1). The result of hardness peak over the tip radius is given in Fig. 5b. The results from different approaches seem follow the same trend, a power-law fitting curve to the data is also plotted in the figure. The simulation and experiment results overlap at R = 100 nm, although the hardness is higher from experiment. The hardness curves in terms of relatively depth (h/R) are plotted in Fig. 5c and d, it can be observed that when h/R is small, the curves coincide with each other, since the indentation process is still in elastic regime, and hardness is in proportion to $\sqrt{h/R}$ according to Hertzian theory^21^. The hardness transition occurs in spherical part of the indenter, as the hardness peaks are on the left side of the green line which indicates the boundary of spherical and conical part of the indenter.

All the results obtained in this paper (*R* = 100 nm) and the relevant ones in literature are plotted in Fig. 1. After considering the tip radius, the hardness obtained from our simulations and experiments show the same trend with the indentation depth, and are in the same order as well. Since Noreyan’s MD simulations did not consider the radius of the tip, it is understandable that his results showed much higher hardness. Also, Ngan’s experimental results showed the similar trend as that reported here but with some difference in the magnitude. We suppose, it should be attributed to the difference in the tips used, since we have already known the sensitivity of nano-hardness to the tip.

Finally, we note that there are mismatches in the results from simulations and experiments, in load and hardness curves in Fig. 4, and the depth of hardness peak in Fig. 4a and Fig. 5c and d. The difference of load and depth to reach the hardness peak between the simulation and experiment is caused the strain rate gap between simulation and actual experiments, as the MD simulation is with a higher strain rate compares to the quasi-static loading in experiment, the load obtained in MD simulation will be higher than that from the experiments. On the other hand, the hardness value is lower from the simulation, which is caused by the differences in definition of the contact area. In this study, the contact area is defined as the projected area of all the carbon atoms in repulse force range, normal to the indenter, which can be easily computed in the simulation. However, this will cause an overestimate of the contact area, since when the total force on the indenter is zero (when total attraction force cancels out to the total repulse force on the indenter, see in Supplementary Fig. 8), there will be a certain contact area calculated based on the repulse force components. Since the attraction force will be larger for larger tip radius, the total repulse force will be larger as well, which results in a larger contact area at the beginning of the indentation process. Thus the hardness from the simulation is lower. The trends of hardness in terms of the depth and indenter tip radius are not affected by these differences.

By combining and cross validation of the large scale MD simulations and experiments, the hardness of Cu is confirmed in this study, as hardness increases then decreases when indentation depth decrease, which indicates that the conventional size effect of hardness is not valid in the range of a few nanometers, it highly depends on the radius of the indenter tip. Larger tip radius will results in a lower hardness peak value. Displacement “bursts” patterns in load-depth curves can be observed in experiments and simulations, which results in the hardness to drop, and they are related to various dislocation events.

## Methods

### Material preparation

The surface of copper specimen of 5 × 5 × 1 mm (Supplementary Fig. 1) was mechanical finished first then followed by electropolish to remove 20 microns surface layer, it was examined by AFM probes and SEM before indentation tests to ensure the surface roughness was within 5 nm and no apparent grain boundaries.

### Indentation testing

Indentation tests were performed using Agilent Nano Indenter G200, and the contacted area was calibrated on fused silica sample to a maximum depth of 350 nm before test on copper specimens. For each depth and load, at least 20 tests were performed to see if the results converged. CSM option was adoped to form a modulus/hardness-depth curve from a single test, with strain rate of 0.05 s^−1^, and 1.0 nm harmonic displacement.

### SEM characterization and EBSD analysis

Zeiss Merlin Compact field emission SEM with Electron Backscatter Diffraction (EBSD) was employed to analyze on crystalline orientations and the tip radius of the indenters. The misorientation (see in Supplementary Fig. 1) to the presumed crystalline direction was within 1.5 degrees for all the specimens of three directions (within 0.5 degree for (100), within 0.8 degree for (110) and within 1.5 degree for (111)). SEM image of the tip from one project direction was taken and from which the tip radius was obtained Supplementary Fig. 2), for Tip-100, 95.9 nm and Tip-150, 151.4 nm.

### AFM characterization

Veeco MultiMode™ AFM with TESP-SS probe of 2 nm tip radius on tapping mode was used for surface roughness and the 3D morphology of indenter tip. The surface roughness (*Ra*, *Rq* and *Rt*) of the sample was calculated from the obtained surface morphology, the results shown that the roughness was in nm scale (Supplementary Fig. 1 and Table. I). Through 3D-reconstructed of the tip, the tip radius of indenter was deduced to be approximate 100 nm and 150 nm (Tip-100, 108.0 ± 5.3 nm and Tip-150, 154.3 ± 13.6 nm).

## Electronic supplementary material

Supplementary document
