# Supplementary material for: Two opposite size effects of hardness at real nano-scale and their distinct origins
Source: Sci Rep. 2017 Nov 22;7:16053. doi: 10.1038/s41598-017-14734-w (PMC5700042; doi:10.1038/s41598-017-14734-w)
Supplement: Supplementary file 1 — Supplementary document [file 41598_2017_14734_MOESM1_ESM.pdf]

Two opposite size effects of hardness at real nano-scale and their distinct origins

Rong Yang, Qun Zhang, Pan Xiao, Jun Wang & Yilong Bai

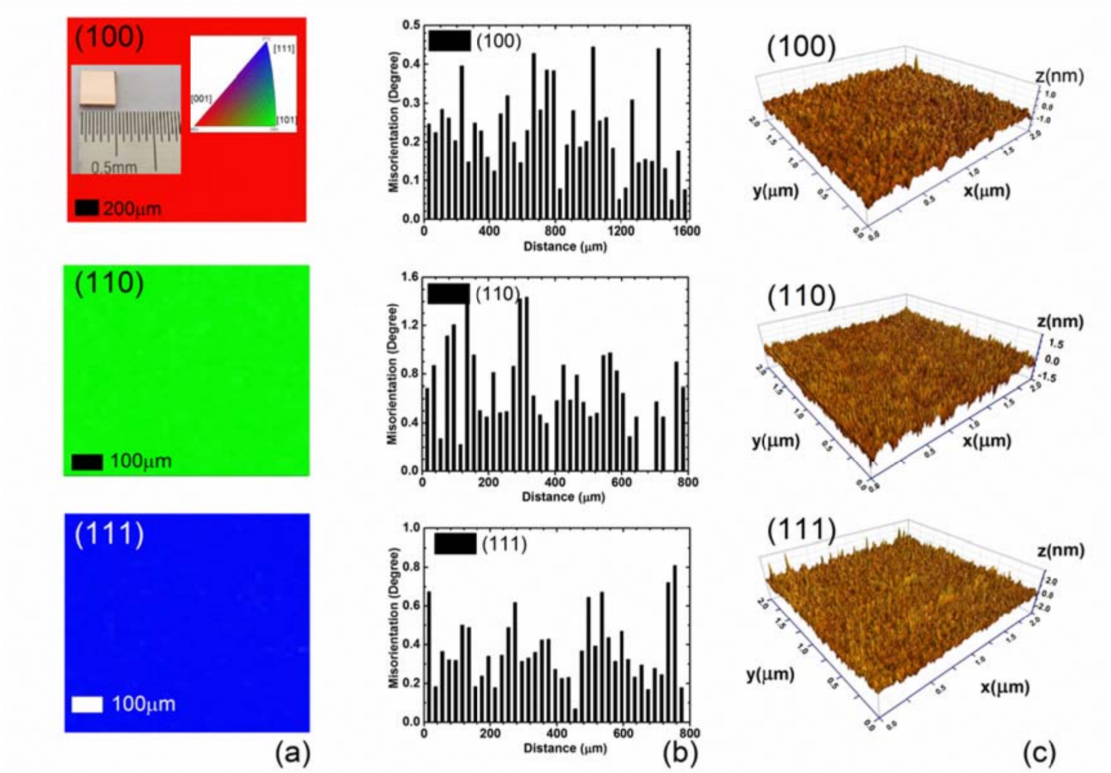

Supplementary Figure 1| Specimen specifications. (a) EBSD map for the surface orientation of (100), (110) and (111), no apparent grains and grain boundaries are observed. (b) Misorientation profiles of the surface to the presumed orientations, the misorientations are within 1.5 degree. (c) Surface morphology of specimens, scan size of 2×2 μm, the roughness peaks are under 3 nm.

Surface	*Ra* (nm)	*Rq* (nm)	*Rt* (nm)
(1 0 0)	0.170	0.216	1.98
(1 1 0)	0.201	0.256	2.10
(1 1 1)	0.261	0.348	3.19

Supplementary Table I| Roughness of the specimen surface. *Ra* is the average roughness (arithmetic average), *Rq* is the root-mean-square roughness and *Rt* is the vertical distance from the deepest valley to the highest peak.

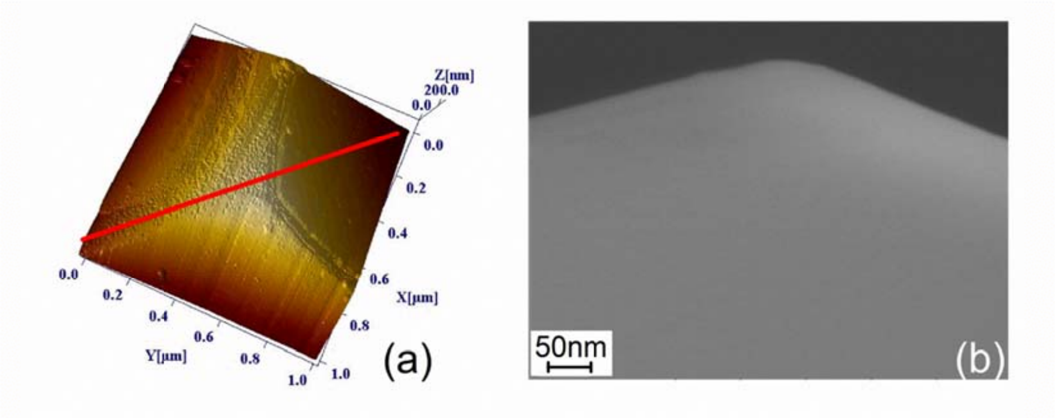

Supplementary Figure 2| Shape of indenter for Tip-150. (a) 3D morphology of tip from AFM result; (b) SEM image of the tip from one project direction.

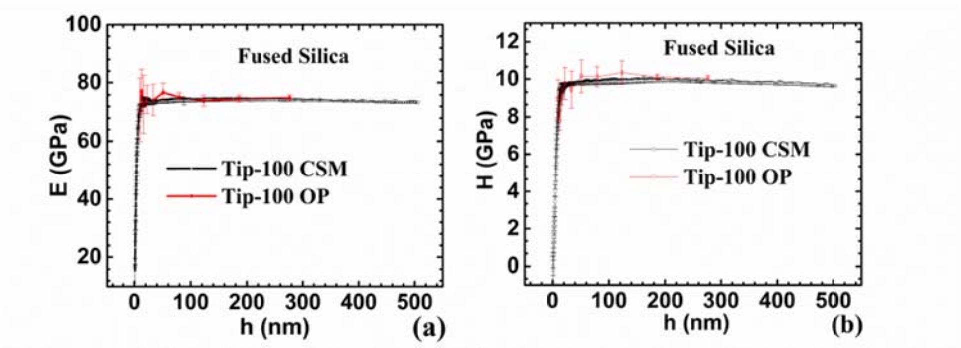

Supplementary Figure 3| IIT test results of modulus and hardness over the depth on fused silica. No apparent size effect was observed on modulus and hardness. (a) modulus of Fused Silica over depth; (b) hardness of Fused Silica over depth.

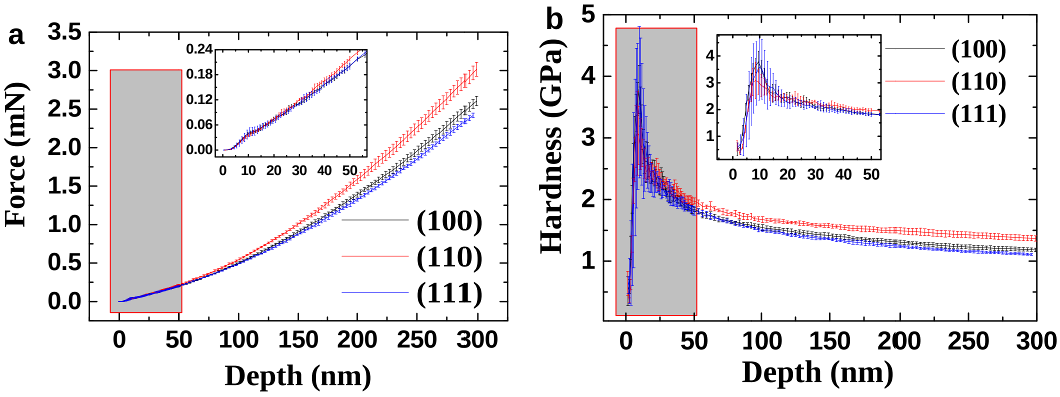

Supplementary Figure 4| Experiment results of force and hardness curves of three orientations on Cu. (a) Force over depth. At the same depth, $F_{(110)} > F_{(100)} > F_{(111)}$; (b) Hardness over depth. At the same depth, $H_{(110)} > H_{(100)} > H_{(111)}$.

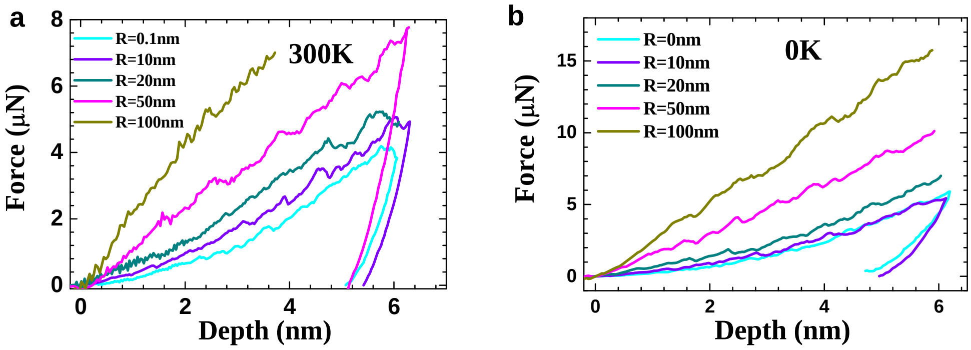

Supplementary Figure 5| Force curves from simulation for different tip radius, the load is higher at 0 K. (a) At 300 K. (b) At 0 K.

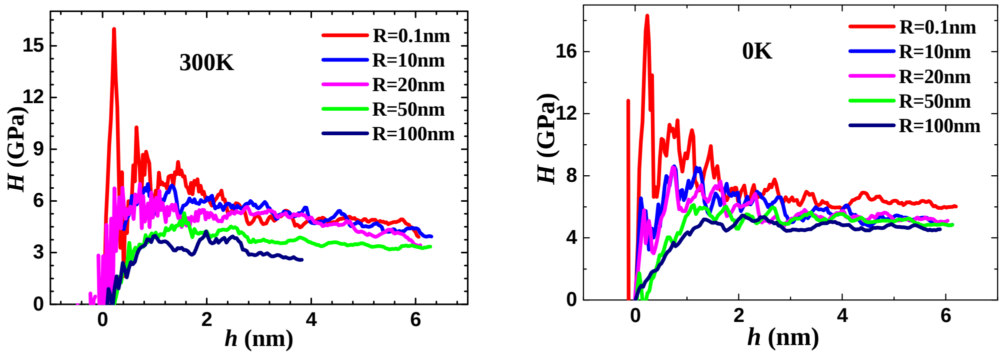

Supplementary Figure 6| Hardness curves from simulation for different tip radius, the hardness is higher at 0 K. (a) At 300 K. (b) At 0 K.

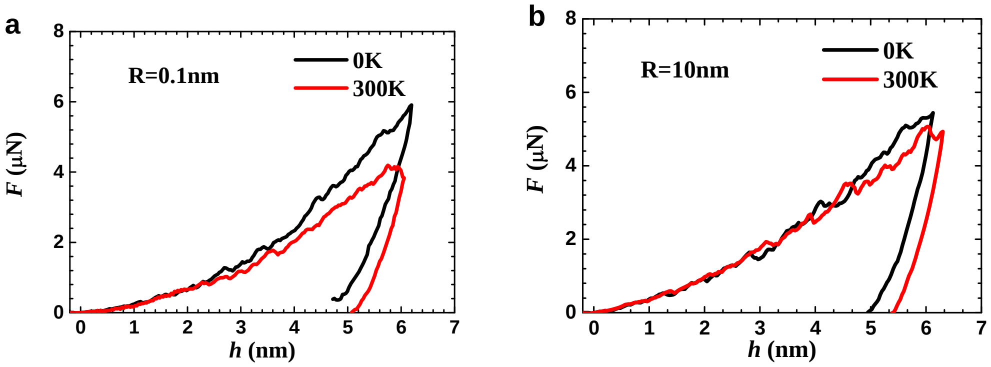

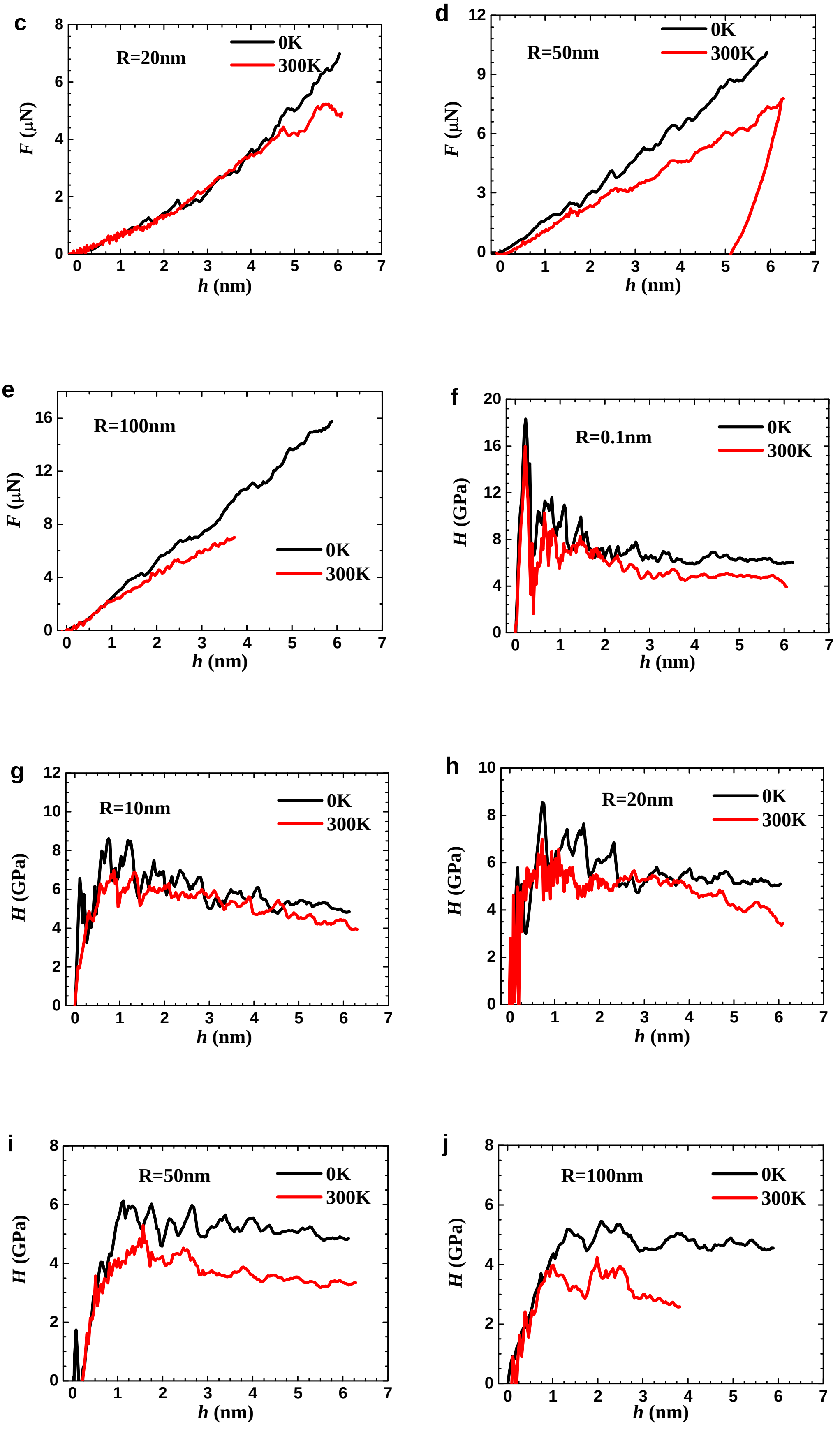
c
R=20nm
0K
300K
F (μN)
h (nm)
d
R=50nm
0K
300K
F (μN)
h (nm)
e
R=100nm
0K
300K
F (μN)
h (nm)
f
R=0.1nm
0K
300K
H (GPa)
h (nm)
g
R=10nm
0K
300K
H (GPa)
h (nm)
h
R=20nm
0K
300K
H (GPa)
h (nm)
i
R=50nm
0K
300K
H (GPa)
h (nm)
j
R=100nm
0K
300K
H (GPa)
h (nm)

Supplementary Figure 7| Comparison of force, hardness between the Hardness curves for different tip radius in MD simulation, the force and hardness are higher at 0 K for each radius. (a) Force for R = 0.1 nm. (b) Force for R =10 nm. (c) Force for R =20 nm. (d) Force for R =50 nm. (e) Force for R =100 nm. (f) Hardness for R = 0.1 nm. (g) Hardness for R =10 nm. (h) Hardness for R =20 nm. (i) Hardness for R =50 nm. (j) Hardness for R =100 nm.

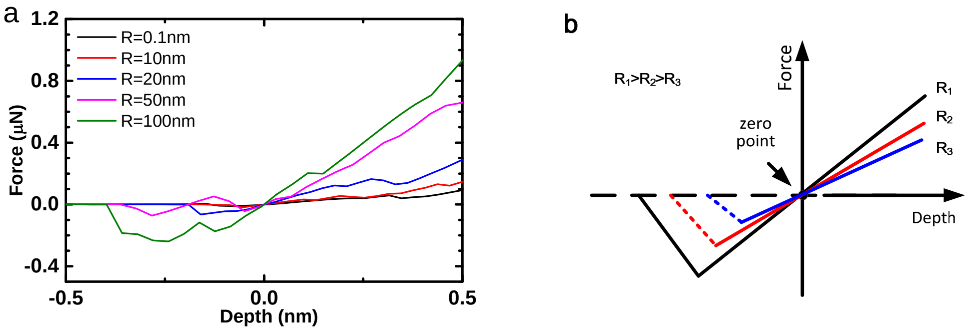

Supplementary Figure 8| Zero point of force and depth definition in MD simulation. (a) Results from simulation for different tip radius. (b) Illustration of the force curve. The total force on the indenter transits from attraction to repulse force when the depth increases, and the zero force point is set to be the point when repulse force cancels out the attraction force. When the indenter comes close to the sample surface, the force is consisted of attraction force between the carbon and copper atoms, so the first part of the loading curve is negative, and larger tip radius results in steeper slope. Then some atoms come into the repulse range, the force goes up and passes zero point as the indentation depth increases, but the slope of the curve is lower than the initial part. The slope of the curve from the smaller tip radius is lower than that from larger radius.
